# Are Retrospective Assessments Means of People’s Experiences? Accounting for Interpersonal and Intrapersonal Variability when Comparing Retrospective Assessment Data to Ecological Momentary Assessment Data

**DOI:** 10.17505/jpor.2022.24855

**Published:** 2022-12-22

**Authors:** IJsbrand Leertouwer, Noémi K. Schuurman, Jeroen K. Vermunt

**Affiliations:** 1 Department of Methodology and Statistics, Tilburg University, Warandelaan 2 PO Box 90153, 5000 LE Tilburg, The Netherlands; 2 Department of Methodology and Statistics, Utrecht University, Heidelberglaan 1, 3584 CS Utrecht, The Netherlands

**Keywords:** Ecological Momentary Assessment, retrospective assessment, retrospective bias, interpersonal variability, intrapersonal variability, affect data

## Abstract

Retrospective Assessment (RA) scores are often found to be higher than the mean of Ecological Momentary Assessment (EMA) scores about a concurrent period. This difference is generally interpreted as bias towards salient experiences in RA. During RA participants are often asked to summarize their experiences in unspecific terms, leaving room for personal interpretation. As a result, participants may use various strategies to summarize their experiences. In this study, we reanalyzed an existing dataset (*N* = 92) using a repeated *N* = 1 approach. We assessed for each participant whether it was likely that their RA score was an approximation of the mean of their experiences as captured by their EMA scores. We found considerable interpersonal differences in the difference between EMA scores and RA scores, as well as some extreme cases. Furthermore, for a considerable part of the sample (*n* = 46 for positive affect, *n* = 56 for negative affect), we did not reject the null hypothesis that their RA score represented the mean of their experiences as captured by their EMA scores. We conclude that in its current unspecific form RA may facilitate bias, although not for everyone. Future studies may determine whether differences between RA and EMA are mitigated using more specific forms of RA, while acknowledging interindividual differences.

## Introduction

In an *Ecological Momentary Assessment* (EMA) data collection procedure, participants repeatedly report on their moment-to-moment experiences in their own environment. One of the supposed key merits of EMA data is that they are more objective than *Retrospective Assessment* (RA) data, in which people retrospectively report on their experiences (cf. Kahneman & Riis, 2005; Shiffman et al., 2008). The reasoning behind this claim is that EMA measurements are close to actual experience both in time and in simplicity (e.g., *“How excited do you feel right now?”*), while RA measurements require participants to recall and summarize their past experiences (e.g., *“How excited did you feel last week?”*), which may be prone to biases and heuristics.

Taken together, these biases and heuristics often entail that salient experiences and recent experiences have a higher probability of being recalled, and are overrepresented in the retrospective summary that people make of their recalled experiences (cf. Bower, 1981; Fredrickson, 2000; Fredrickson & Kahneman, 1993; Kahneman et al., 1993; Redelmeier & Kahneman, 1996; Tversky & Kahneman, 1973). Supporting evidence that psychological biases towards salient experiences drive the difference between RA and EMA comes from studies in which participants’ RA summary score for their affective experiences was found to be higher than a summary of their EMA scores (cf. Ben-Zeev et al., 2009; Colombo et al., 2020; Ebner-Priemer et al., 2006; Kardum & Daskijević, 2001; Lay et al., 2017; Neubauer et al., 2020; Parkinson et al., 1995; Rinner et al., 2019; Thomas & Diener, 1990; Wenze et al., 2012).

However, methodological aspects of these studies may influence the extent to which psychological biases and heuristics influence the difference between RA and EMA. In this study, we will investigate one such methodological aspect: a possible mismatch between summary statistics in EMA and RA. Specifically, in many but not all studies that compare RA to EMA, the EMA data are summarized by their mean, while during RA participants are asked to summarize their recalled experiences using a summary statistic that is *unspecific* (e.g., *“How excited did you feel last week?”*; exceptions are Kardum & Daskijević, 2001; Thomas & Diener, 1990). Although not made explicit, it appears that the mean is often chosen as a summary statistic for EMA because it ascribes equal weight to all recorded experiences, and is thus *‘most fair to all experiences’* (i.e., unbiased). In contrast, when participants are asked in general terms to what extent they had experienced a certain experience during the timeframe under consideration, this appeals to the summary that people personally make by default. Such a personal default summary may be especially prone to biases and heuristics. The expectation based on the results of these comparison studies can be summarized as follows:


unspecific(remembered experiences) > mean(EMA experiences) (1)

where *unspecific*() represents the unspecific summary function that a participant is required to apply during RA; *remembered experiences* is the subset out of all the participant’s experiences that they recall during RA to which they apply their unspecific summary score function; *mean*() represents the mean that is used to summarize the EMA data; and *EMA experiences* represents the subset out of all the participant’s experiences that is gathered through EMA. In Equation (1), the inequality signifies that it is generally reported that RA scores (i.e., the part before the > sign) are higher than the means of their respective EMA scores, supposedly because of biases in the unspecific summary function and subset of remembered experiences invoked by RA.

Note that if participants were specifically instructed to estimate the mean of an experience during RA (e.g., *“how excited on average did you feel last week?”*; c.f., Kardum and Daskijević, 2001), and this summary would be compared to the mean of their EMA data, only the subsets would differ between assessment methods.^1^ In the frequently used unspecific form of RA, however, the summary function that people apply (as well as the subset of recalled experiences) can take many different forms. For example, some people may actually try to approximate the mean of their recalled experiences, while others may tend to ascribe stronger weight to salient or recent experiences. Supporting this notion, some authors report high interpersonal variability in the difference score between EMA means and unspecific RA summary scores (Neubauer et al., 2020).

In addition to their use of an unspecific RA summary, a characteristic of current studies in which RA data are compared to EMA data is that they exclusively use point estimates: the RA summary score (i.e., typically unspecific) and the EMA summary score (i.e., typically the mean). However, there may be untapped information in the intrapersonal variability in the EMA data. For example, when a participant’s RA summary score (e.g., 4) lies outside of the range of their recorded EMA data (e.g., 1-3), this formally rules out that the participant used the mean to summarize their experiences as captured by their EMA data.

In this study we aim to learn more about the summaries that people make when they are asked to make an unspecific RA summary of their experiences, by considering how their EMA scores are distributed. To this end, we will reanalyze an existing dataset (Colombo et al., 2020). We are specifically interested in falsifying or corroborating that as their RA summary score, participants used an approximation of the mean of their experiences as captured by their EMA data. As such, whereas previous authors used a top-down approach to establish a difference between the mean of participants EMA data and their unspecific RA summary score, we will use a bottom-up (repeated *N* = 1) approach: we will consider the difference between the unspecific RA summary score and the distribution of EMA scores per individual, to make a qualitative decision about whether their unspecific RA summary may be an approximation of the mean of their experience as measured by their EMA data. Our specific interest in the mean is driven by the fact that it is the most widely used statistic to summarize EMA data in the EMA literature, in studies that compare different data types as well as other studies. Our analyses will give an indication of whether participants also tend to summarize their experiences by the mean value when they are free to use any summary statistic. By considering the data for each participant separately, we fully take into account that there may be interindividual differences in the summary that participants make during RA.

Our analyses are explorative; based on the reported standard deviations and results of cluster analyses in the original manuscript (Colombo et al., 2020), and results of a similar study (Neubauer et al., 2020) we expected to find interpersonal differences in the difference scores between EMA means and RA summary scores. Other than this broadly formulated expectation, we did not have any formal hypotheses. In the following sections, we will first describe the original dataset, before we explain our complementary analyses about the interpersonal and intrapersonal variability in the data.

## Methods

### Dataset & Original study

The dataset was originally used by Colombo et al. (2020) to study whether discrepancies between EMA means and RA summary scores were related to self-reported mental health and resilience. Participants were undergraduate students (*N* = 92) who scored below a threshold for general pathology and Generalized Anxiety Disorder pathology (Patient Health Questionnaire-9; Kroenke et al., 2001; Generalized Anxiety Disorder pathology-7; Spitzer et al., 2006; combined score below 14). During the EMA data collection, they reported on one positive and one negative affect item three times per day for 14 days. The items had 5-point Likert scales (1 = *“not at all”*; 5 = *“extremely”*) and were phrased as: *“To what extent are you experiencing positive [-negative] emotions at this moment?”*. After the EMA data collection, participants filled in a Spanish version (Díaz-García et al., 2020) of the Positive and Negative Affect Schedule (PANAS; Watson et al., 1988), in which they considered the extent to which they experienced 10 positive affective experiences and 10 negative affective experiences during the previous 14-day period (range 1-5; referring to *“very slightly or not at all”, “a little”, “moderately”, “quite a bit”*, and *“extremely”* respectively). For each participant the mean scores of the 10 positive and the 10 negative affect items of the PANAS were calculated. In the original study, these two resulting RA summary scores of positive and negative affect were then compared to the means of the positive and negative affect EMA item scores.

Colombo et al. (2020) used Wilcoxon Signed Ranks Tests on the *raw difference scores* between the two RA summary scores and the two EMA means (i.e., the RA summary score minus the EMA mean), and found that on *group-level*, the RA summary scores were higher than the EMA means for negative affect, but not for positive affect.^2^ In our analyses, we extend the information on these raw difference scores by reporting in detail on how they varied over *individuals*. Most importantly, we focus on a new aspect of the data: the intrapersonal variability in the EMA data per individual.

### Analyses

#### Raw difference scores

We started by inspecting the interpersonal differences in the raw difference scores between the EMA means and the RA summary scores. From the original manuscript, we learned that the mean difference score for negative affect (i.e., the RA summary score minus the EMA mean) was found to be significantly higher than 0, while for positive affect this was not the case. We additionally provide and evaluate the distribution of raw difference scores across participants. This provides us with information about the exact variability in the difference scores, and whether there are any outliers.

We calculated the means for the EMA data per participant based on their observed scores, excluding any missing observations. There were some discrepancies between our calculated means and those of the original authors. Based on these discrepancies, we decided to exclude one participant for which we found a difference of a full point with the originally reported data. For the other participants, the differences were small and we used the mean values that we calculated.^3^

An important characteristic of the EMA data was that the number of complete observations varied, with 19 participants (21%) completing less than 27 questionnaires (65% of all questionnaires, which was the threshold for receiving a reward for participation in the original study; see Appendix B for details). The number of completed questionnaires is an important characteristic of the EMA data, as it may impact the extent to which the subset of experiences captured by EMA is representative of all experiences that participants had.

For the RA summary scores, we adopted the values reported by the original authors, given that we did not have access to the separate PANAS scores per participant. An important consideration for these RA summary scores of positive and negative affect (range 1-5) is that they were the mean of 10 items per affect valence in the PANAS, while the EMA means were based on general ratings of *“negative emotions”* or *“positive emotions”*. As such, like the original authors, we need to assume that the 10 items for positive and negative affect described in the PANAS match the general affective experiences that participants reported about during EMA. We will return to this assumption in the discussion section of this manuscript. Finally, we subtracted the EMA means from the RA summary scores to get the raw difference scores for both positive affect and negative affect.

#### Variability in EMA data

In the second part of the analyses, we considered the intrapersonal variability in the EMA data, which is the variability of the EMA scores per participant. To summarize the intrapersonal variability in the EMA data for positive affect and negative affect, we calculated means, modes, interquartile^4^ and full ranges per participant, and reported means, standard deviations and histograms for these statistics.

Then, we determined how many RA summary scores fell within their respective interquartile- and full range of EMA data for positive affect and for negative affect. RA summary scores that fall outside of the range of EMA data are particularly interesting as in this case there is the clearest mismatch between EMA mean and RA summary score.^5^

#### Optimized distributions for each participant

Next, we created two probability distributions for each participant for both positive and negative affect. An observed probability distribution based on their EMA data, and an optimized probability distribution based on their RA summary score. The observed probability distribution of a participant (henceforth *“observed EMA distribution”*) contains estimates of the probabilities of having an affect score with intensity 1 to 5 during the period of investigation, based on their EMA data. The optimized probability distribution based on participants’ RA summary score (henceforth *“optimized RA distribution”*) represents a hypothetical distribution of recalled experiences that is as similar to the set of experiences captured by EMA as possible, given that the RA summary score is the mean of this distribution of recalled experiences. If these probability distributions are highly similar, we conclude that it is not unlikely that the RA summary score represents the mean of experiences as captured by EMA.

To create the observed EMA distribution, we divided the number of EMA observations per answer category by the total number of EMA observations for each participant: This provides us with the relative frequency for each answer category. To create the optimized RA distribution, we used two specifications. First, the RA summary score should represent the mean of the optimized distribution. Second, the optimized distribution should be as similar as possible to the observed EMA distribution. Specifically, we used the observed EMA distribution for each participant as a starting point. Then, we used an optimization algorithm to minimize the log-likelihood difference between the observed EMA probability distribution and the optimized RA probability distribution under a mean restriction. The mean restriction that we specified was the RA summary score. For these analyses, we used the *NlcOptim package* (Chen & Yin, 2016) in the *R* programming environment (R Core Team, 2021). Details of this procedure are described in Appendix A.

#### Differences between observed and optimized distributions for each participant

To test whether the observed EMA distribution was significantly different from the optimized RA distribution for each participant for each affect, we used chi-squared tests. The null hypothesis for this difference test is that the observed EMA distribution is identical to the optimized RA distribution. A failure to reject the null hypothesis means that there is no evidence that the participant did not use the mean of their experiences as captured by EMA to retrospectively summarize their experiences of the past 14 days. A rejection of the null hypothesis comes with various possible interpretations: First, the summary statistic that the participant used as their RA summary may be different from the mean. Second, the subset of remembered experiences that the participant applies their RA summary to differs from the subset of experiences recorded with EMA. Finally, the participant’s RA summary score may be qualitatively different from their EMA data. We will return to these interpretations in the discussion section of this manuscript.

We also evaluated the effect size of the differences between the observed EMA distributions and optimized RA distributions per participant, using the Jensen-Shannon divergence (JSD; Lin, 1991; Lin & Wong, 1990; Wong & You, 1985). The JSD expresses the combined distance between two distributions and their average distribution. It ranges from 0 to 1, where 0 indicates that distributions are identical, and 1 indicates that distributions are completely different (for more details, see Appendix A).

#### Explorative analyses of extreme cases

In the final part of our analyses, we considered two explanations for extreme differences that we detected between the EMA data and RA data for a number of participants. The first explanation that we considered was that the subset of experiences captured by EMA may not be representative of the participant’s actual experiences. Although we do not have access to the participant's true experiences to verify this, we did have information on their number of completed questionnaires. We checked whether extreme cases completed few questionnaires, given that this may negatively impact the extent to which their EMA data are representative of all their experiences.

The second explanation that we considered was that instead of the mean, participants used a specific other RA summary: the *peak-end rule* (cf. Ben-Zeev et al., 2009; Fredrickson, 2000; Fredrickson & Kahneman, 1993; Ganzach & Yaor, 2019; Kahneman et al., 1993; Redelmeier & Kahneman, 1996; Lay et al., 2017). According to authors studying this summary, people tend to summarize their experience by taking the average of the most intense moment and the final moment of the experience. In line with some previous authors (Lay et al., 2017), we calculated the peak-end rule summary by taking the mean of the last three EMA scores on the last day of the EMA data collection and then taking the mean of both this calculated mean and the highest EMA score. We chose to take the mean of the three EMA scores of the last day rather than the very last measurement to get a more stable affect score for the final stage of the (relatively long) period of investigation. To get the raw difference score, we then subtracted our peak-end rule summary score from participants’ RA summary score for positive and negative affect.

## Results

### Raw difference scores

The means of the raw difference scores between RA summary scores and EMA means based on our calculations were .058 (*SD* = 0.84) for positive affect and 0.415 *(SD* = 0.56) for negative affect.^6^ In other words, the mean difference score for positive affect was almost equal to zero, and for negative affect the mean difference score was less than half a point. In addition, the variability in raw difference scores was larger for positive affect than for negative affect.

**Figure 1:**
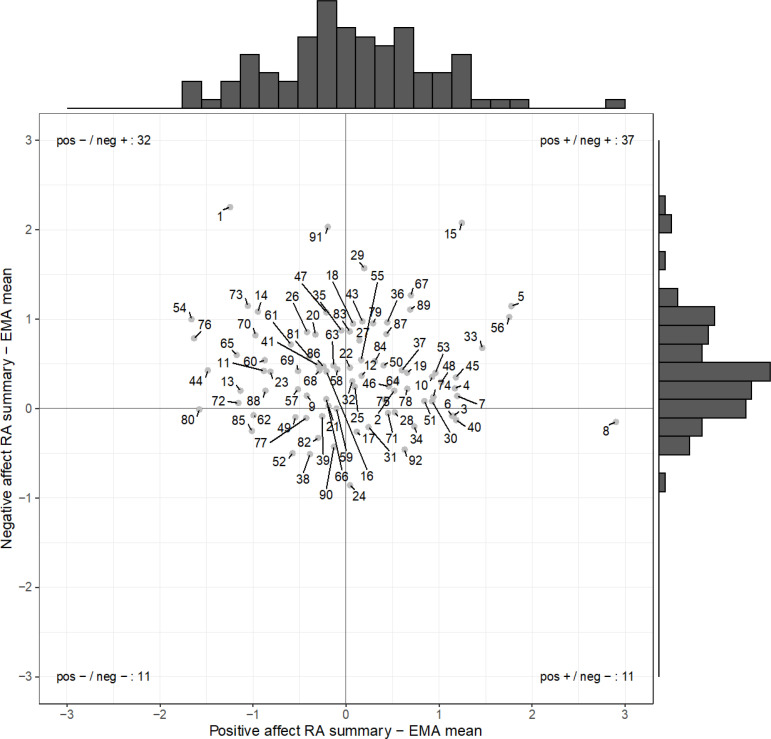
Raw difference scores between the RA summary scores and the mean of the EMA data for positive affect (x-axis) and negative affect (y-axis). Each point represents a participant; the corresponding numbers represent ID numbers. The horizontal and vertical dark lines mark a raw difference score of 0. Positive scores reflect higher retrospective scores, while negative scores represent that the mean of the EMA data is higher. The histogram above the plot area shows the distribution of raw difference scores for positive affect, and the histogram right of the plot area shows the distribution of raw difference scores for negative affect. The number of observations within each quadrant is printed in the corners of the plot.

In order to get a complete overview of the raw difference scores, we plotted them together in Figure 1. From the histograms in Figure 1 it is also clear that there is more variability in the difference scores for positive affect than for negative affect. Furthermore, based on this plot we see that three participants have a difference score that is higher than two full points for negative affect (ID = 1, 15, 91), and that one participant has a difference score higher than two full points for positive affect (ID = 8). We will return to these extreme cases in the final paragraph of this section.

As can be observed from the scatterplot Figure 1, there was no relation between the raw difference scores for positive and negative affect (*r* = -.06. for raw difference scores; *r* = .01 for absolute difference scores). Concerning the relation between raw difference scores for positive and negative affect, Figure 1 also shows that all four combinations of the valence of raw difference scores occur. That is, we see that some participants’ RA summary score was higher than the mean of their EMA data for both positive affect and negative affect (top right quadrant of Figure 1, *n* = 37), while other participants’ RA summary score was lower than the mean of their EMA data for both positive affect and negative affect (bottom left quadrant of Figure 1, *n* = 11). Participants with a higher RA summary than the mean of their EMA data for negative affect combined with a lower RA summary than the mean of their EMA data for positive affect also occurred (top left quadrant of Figure 1, *n* = 32), as did participants with a lower RA summary score than the mean of their EMA data for negative affect, combined with a higher RA summary score than the mean of their EMA data for positive affect (bottom right quadrant of Figure 1, *n* = 11). In conclusion, there were considerable interindividual differences in the raw difference scores between the RA summary scores and EMA means, in terms of size and valence.

### Variability in EMA data

Next, we considered how EMA scores were distributed per participant for positive and negative affect. These observed EMA distributions are presented as histograms in Figures 2a-b (printed in green in Figure 2a for positive affect, and printed in blue in Figure 2b for negative affect). In these figures, answer options are found on the x-axes and the relative frequency (probability) of the response is found on the y-axes. What stands out from these figures is the interindividual variability in the shape of the EMA distributions. Furthermore, with respect of the combination of EMA distribution and RA summary score, some participants show high intrapersonal variability in EMA data combined with an RA summary score that is close to the mean (e.g., Figure 2a, ID = 18). Others show low intrapersonal variability in EMA data combined with an RA summary score that is far from the mean (e.g., Figure 2b, ID = 15), and there are many configurations in between. Hence, taking the intraindividual variability into account when assessing the difference between EMA data and RA summary scores should provide us with additional information.

**Figure 2a:**
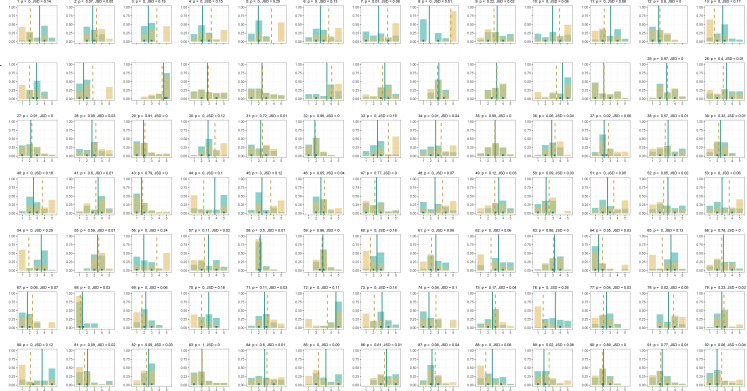
Distributions for positive affect for all participants. The green bars are the observed probabilities for the different category scores. The solid vertical green lines represent the observed mean based on the EMA data, and the short green lines mark the interquartile range. The dashed vertical yellow line represents the RA summary. The yellow bars represent the optimized probabilities under a mean constraint that is equal to the retrospective summary score. Above each plot, the participant’s ID number is printed first, followed by the p-value of the chi-squared test between the two distributions (p) and the Jensen-Shannon divergence between distributions (JSD).

**Figure 2b:**
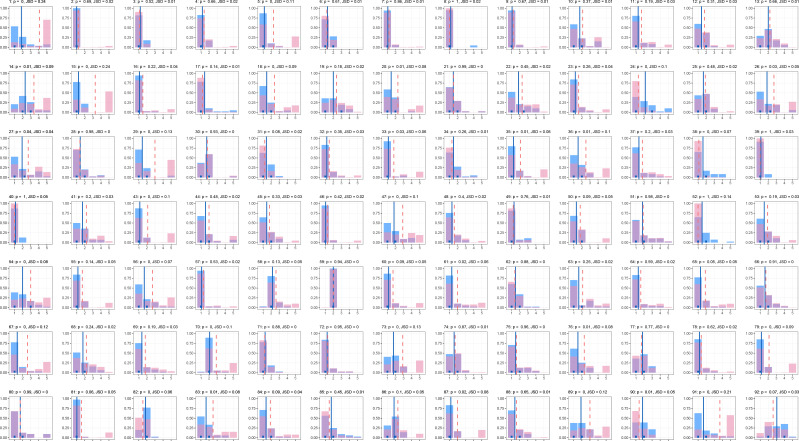
Distributions for negative affect for all participants. The blue bars are the observed probabilities for the different category scores. The solid vertical blue lines represent the observed mean based on the EMA data, and the short blue lines mark the interquartile range. The dashed vertical pink line represents the RA summary score. The pink bars represent the optimized probabilities under a mean constraint that is equal to the retrospective score. Above each plot, the participant’s ID number is printed first, followed by the p-value of the chi-squared test between the two distributions (p) and the Jensen-Shannon divergence between distributions (JSD).

**Figure 3:**
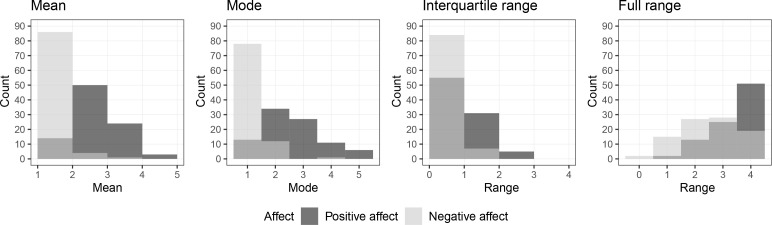
Histograms for the mean, mode, interquartile -and full range of EMA data per participant for EMA measurements of positive affect (dark grey) and negative affect (light grey).

The mean, mode, interquartile -and full range of the EMA data for positive affect and negative affect scores across participants are presented in Table 1, and histograms for these summary statistics can be found in Figure 3. For many participants, the EMA scores for negative affect had a low mean, mode, and interquartile range, indicating that most participants report experiencing relatively little negative affect in their EMA scores. In comparison, the EMA scores for positive affect across participants are more symmetrically distributed around midpoint scores, with considerably more variability.

**Table 1:** Means and standard deviations of the mean, mode, interquartile - and full range of the EMA data per participant.

Statistic	Positive affect	Negative affect
Mean	2.70 (0.66)	1.51 (0.35)
Mode	2.59 (1.09)	1.16 (0.45)
Interquartile range	1.35 (0.65)	0.61 (0.59)
Range	3.37 (0.81)	2.52 (1.10)

*Note.* Values in brackets are standard deviations.

When we compared the participants’ RA summary scores to their interquartile -and full range in EMA scores, we found that for positive affect 42 out of 91 RA summary scores fell within their respective interquartile range and for 87 RA summary scores fell within their respective full range of EMA scores. For negative affect, 27 RA summary scores fell within their respective interquartile range, and 87 fell within the full range. We will discuss cases for which the RA summary fell outside of the range of their EMA data in the final paragraph of this section.

### Optimized distributions

The optimized distributions can also be found in Figures 2a-b (printed in yellow in Figure 2a for positive affect, and printed in pink in Figure 2b for negative affect). A characteristic of the optimized RA distributions is that when the difference between the observed EMA distribution and the RA summary score is large, the algorithm tends to compensate through ascribing high probability to the highest (e.g., Figure 2a, ID = 33) or lowest (e.g., Figure 2a, ID = 65) category scores, rather than ascribing even higher probability to category scores that are closer to high observed probabilities. Although it is debatable whether this characteristic is desirable, it does come with a substantive interpretation that relates to established biases. That is, in case of a large difference between the RA summary score and the EMA data, the algorithm takes into account that extreme scores (i.e., salient experiences) should carry particular weight (i.e., probability), rather than scores that are marginally higher than the experiences captured by EMA.

### Differences between observed and optimized distributions

When we compared the optimized RA distributions to the observed EMA distributions, we found that for positive affect, 45 participants had a statistically significant chi-squared test (*p*-value < .05), and for negative affect, a maximum of 35 participants had a statistically significant chi-squared test (see Appendix B for details). For these participants, the null hypothesis that they used the mean of their experiences as captured by EMA to make a retrospective summary during RA was rejected.

The chi-squared tests appeared to be rather strict in concluding that there was a difference between distributions. To illustrate, the biggest difference between distributions (i.e., highest JSD) that was not considered statistically significant was observed for participant 67 for positive affect (JSD = .07, *p* = .06), and the smallest difference that was considered significant was observed for participant 52 for positive affect (JSD = .02, *p* = .05); see Appendix B for details. Note that for many significant chi-squared tests, the distributions actually looked quite similar by visual inspection (see Figures 2a-b).

**Figure 4:**
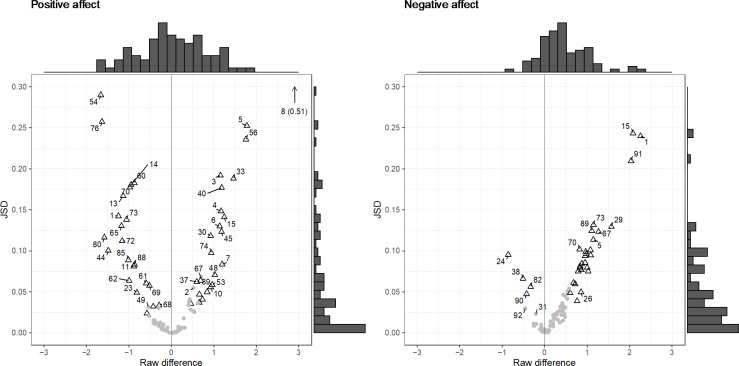
Jensen-Shannon divergence (x-axis) plotted against the raw difference scores (y-axis) for positive affect (left) and negative affect (right). Each point represents a participant; the corresponding numbers represent ID numbers. Triangles represent participants for which the p-value of the chi-squared test was lower than .05.

The relation between the raw difference scores and the effect sizes expressed by the JSD can be found in Figure 4. What stands out from this figure is that the effect sizes were more often higher for positive affect than for negative affect and the distributions of JSDs appeared to be heavily skewed for both positive affect and negative affect. The latter shows that for many participants, the difference between the observed EMA distribution and the optimized RA distribution was relatively small. Note that the highest JSD value was .52 (for positive affect, ID = 8). As the difference between distributions that led to this JSD value seemed extreme (see Figure 2a), we conclude that given the current set-up (i.e., number of responses and answer categories), we do not expect the JSD to span its full range up to 1.

Table 2

**Table 2:** Extreme cases

ID	Extreme	Completed	Positive affect					Negative affect				

			*M*	*Range*	*RA*	*Dif. M*	*Dif. PER*	*M*	*Range*	*RA*	*Dif. M*	*Dif. PER*
3	Outside	.52	2.55	2-3	3.7	1.16	-					
5	Outside	.93	1.92	1-3	3.7	1.78	0.95					
8	Outside	.48	1.80	1-4	4.7	2.90	2.20					
76	JSD	.93	3.33	1-5	1.7	-1.63	0.03*					
54	JSD	.98	3.56	2-5	1.9	-1.66	-0.77*					
56	JSD	.88	1.95	1-4	3.7	1.75	0.70					
15	Outside	.64	1.56	1-2	2.8	1.24	0.97	1.22	1-2	3.3	2.08	1.80
2	Outside	.55						1.00	1-1	1.2	0.20	0.20
43	Outside	.95						1.13	1-2	2.1	0.98	0.60
79	Outside	.98						1.15	1-2	2.1	0.95	0.60
1	Raw	.62						1.85	1-5	4.1	2.25	0.60
91	Raw	.64						1.67	1-5	3.7	2.03	0.20

*Note*. ID = participant’s ID number; Extreme = the analysis based on which the participant was considered extreme (Out = the RA summary fell outside of the range of EMA data; JSD = the JSD value was particularly high; Raw = the raw difference score was particularly high); Completed = the percentage of completed questionnaires, *M* = mean; Range = the range of EMA data; RA is the RA summary; Dif. M = the raw difference between RA summary and EMA mean; Dif. PER = the raw difference between the peak-end-rule summary and the EMA mean.

* Because these participants underestimated their positive affect, we decided to take the average of their lowest score (rather than their highest) and the scores of their final day.

### Explorative analyses of extreme cases

Participants who were considered extreme cases based on the analyses above are listed in Table 4. As is clear from Table 4, approximately half of these participants showed low completion rates, which could indicate that their EMA data were relatively unrepresentative of their experiences, compared to participants with a higher completion rate. Notably, for extreme cases pertaining to negative affect, only participants 43 and 79 showed high completion rates. Their RA summary score fell outside of the range of their EMA data by 0.1 point. Finally, the peak-end rule summary (only) seemed much closer to the RA summary score for participants 1, 76 and 91. For participant 3 we were not able to construct the peak-end summary, as their EMA data for the complete final day were missing.

## Discussion

In this manuscript, we reanalyzed a dataset including EMA data and RA data about a concurrent period, using a bottom-up (i.e., repeated *N* = 1) approach. We complemented the original analyses (Colombo et al., 2020) by zooming in on the interpersonal and intrapersonal variability in the data.

We found considerable interindividual differences in the raw difference scores between RA summary scores and EMA means, as well as some extreme cases. We also observed considerable interpersonal differences in the intrapersonal variability of the EMA data. In addition, an overall pattern was apparent in the intrapersonal spread in the EMA data, in which negative affect data were often distributed over the lowest category scores with low variability, while positive affect scores were often distributed around the middle scores with more variability.

Taking the intrapersonal variability in the EMA data into account when comparing these data to RA data may provide additional information and hypotheses. For example, when we used the intra-personal variability in the EMA data in the comparison with RA data, we found that for a considerable part of participants it was not unlikely that their RA summary score represented the mean of the same distribution of experiences as the observed EMA distribution. This was the case more often for negative affect than for positive affect (*n* = 46 for positive affect, *n* = 56 for negative affect).

There are some noteworthy limitations of the investigated dataset. To start, the dataset contained relatively few observations per participant (*t* = 17-42; see Appendix B for details). This may impact the power of the chi-squared test. However, the chisquared tests seemed strict with regard to establishing a difference (see Appendix B for details). This finding, combined with visual inspection of the two compared distributions indicates that a failure to reject the null hypothesis is indicative of small differences between the distributions. Furthermore, the data were gathered from a relatively small convenience sample of relatively healthy students. Studying a larger community sample, as well as a sample that suffers from psychological problems seems like an important future direction.

Another important consideration for our analyses is that the RA summary scores consisted of the means of specific affective experiences described by the PANAS, while the EMA scores were about general ratings per affect valence. As a result, a difference between the two measures could also be explained by a failure of the PANAS to cover the whole affective spectrum, or a failure of participants to include all affective states mentioned in the PANAS when they were reporting on their affect valences during EMA. In future studies about the difference between EMA and RA, we therefore recommend using the exact same constructs pertaining only to different time indices during EMA and RA. Note in this regard that it can be argued that broad and multifaceted constructs such as general positive and negative affect are unsuited for single-indicator variables in terms of validity and reliability. We encourage future researchers to use specific measurement constructs, and to keep exploring the (concurrent) validity of these constructs. Based on the selected construct(s), reliability may than be determined using either strategies for parallel items (cf. Hu et al., 2016), or single-indicator items (cf. Schuurman et al., 2016; Schuurman & Hamaker 2018).

Another consideration is that we only had a point estimate as an RA summary score to compare to the distribution of EMA experiences. This point estimate RA summary score is a composite measure of the subset of experiences a participant recalls and the summary that they apply to these experiences (i.e., *unspecific(remembered experiences*); see Equation (1)). In our analyses, in essence we reconstructed the subset of remembered experiences to be most similar to the subset of EMA experiences by fixing summary function *unspecific()* to be the mean (i.e., such that we minimize *mean(remembered experiences) - mean(EMA experiences*)). However, because we only have a point estimate to evaluate, we strictly cannot separate the summary function that the participants used from the subset of their remembered experiences. When we fail to find a difference between the observed EMA distribution and the optimized RA distribution, our conclusion applies to both subset and summary. That is, we cannot rule out that the RA summary by the participant applied to their remembered experiences equals the mean of their experiences as captured by their EMA measurements. However, when there is a difference between the observed EMA distribution and the optimized RA distribution, there are several explanations.

First, it may be that participants did approximate the mean of their remembered experiences, but that the subset of remembered experiences was different from the subset of EMA measurements. Although such a difference is often assumed to originate from bias at the retrospective level, it may also result from bias at the level of EMA, most clearly due to missing data. In line with the latter, many participants for which there was the strongest difference between RA and EMA in the sample showed low completion rates.

Second, it may be that the subset of remembered experiences is the same as the subset of EMA experiences, but participants used a summary other than the mean to characterize the subset of remembered experiences. This interpretation does not apply to participants who summarized their subset of remembered experiences by a value that was not recorded at all in their EMA subset of experiences. For these participants, there are again two explanations: the EMA data failed to capture some particularly salient moment(s), and/or these participants re-evaluated the intensity of their experiences at some later point in time. The latter explanation implies that for these participants, the subset of remembered experiences is qualitatively different from the subset of experiences at the moment they were experienced from moment to moment. Note that this may also be the case for participants whose RA summary score did fall within the range of their EMA data. As a final note, from a practical point of view, we cannot rule out that a strong difference between EMA and RA originated from a lack of motivation to participate in one or both parts of the procedure. Determining which interpretation is the correct interpretation when the RA summary score is an unspecific point estimate only seems possible by means of a qualitative study. In such a study, participants would for example be asked specifically how they arrived at their final summary score.

Alternatively, the summary that is specified by RA can be made more specific. For example, by explicitly asking participants about the mean (Kardum & Daskijević, 2001) or some other derivative of their experiences (Thomas & Diener, 1990) during RA, only the subsets of experiences would differ in theory. However, whether participants accurately apply this summary statistic in practice remains unknown in such a design.

Another example of specific RA is to ask participants explicitly to estimate distributions of their experiences (Leertouwer et al., 2021). This procedure should encourage participants to consider experiences of all valences in their subset of remembered experiences. In addition, the distributions for RA and EMA can be compared directly. Finally, this design may be complemented in future studies by also asking for an unspecific point estimate (e.g., *“how excited did you feel last week?”*), or a specific point estimate (e.g., *“how excited did you feel on average last week?”*). This addition would allow for separating the subset (i.e., distribution) of remembered experiences from the point estimate summary function that participants apply to this subset (see equation (1)).

Note that by making the summary more specific, participants are not free in summarizing their past experiences, which makes the construct that is measured different from unspecific RA. Specifically, whereas specific RA may be used to study a difference between RA and EMA data under ideal circumstances, unspecific RA may be used to study a difference between RA and EMA when people make a personal default summary of their experience. Both are interesting areas of research. When more studies become available that make use of a specific form of RA, meta-analyses may determine whether the form of RA (i.e., specific versus unspecific) impacts the difference with EMA measurements.

Studying the difference between EMA and RA is not only important for interpreting studies in which different types of data are compared, but for interpreting any study that includes RA or EMA measurements. With this manuscript we hope to exemplify that although comparison studies of EMA data to RA about affective experiences are relatively unified in their conclusion that RA summary scores are higher than the mean in concurrent EMA measurements across participants, there may be substantial differences in the different data sources from participant to participant. These differences between individuals are important to consider, and deserve further exploration. Our current comparison of distributions generates a range of hypotheses in this context that are worth investigating.

## Figures and Tables

**Figure S1:**
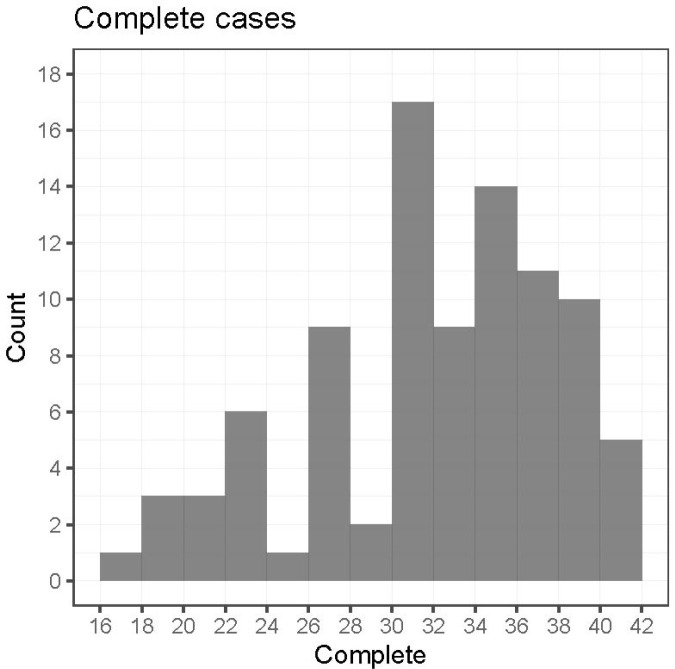
Histogram of the number of completed questionnaires per participant.

**Figure S2:**
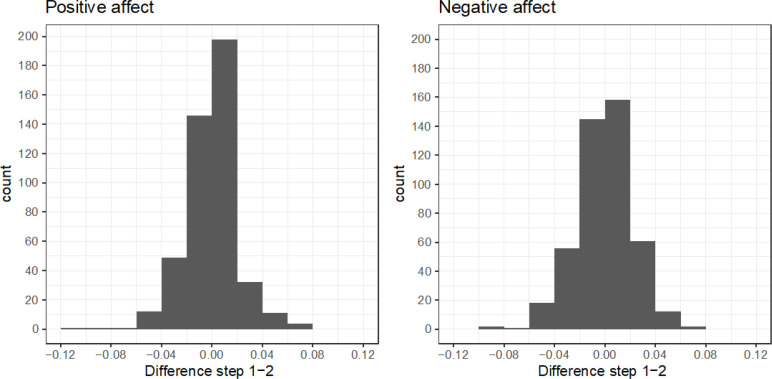
Differences between the estimated probabilities during the first step in the optimization procedure and second step, for positive affect and negative affect.

**Figure S3:**
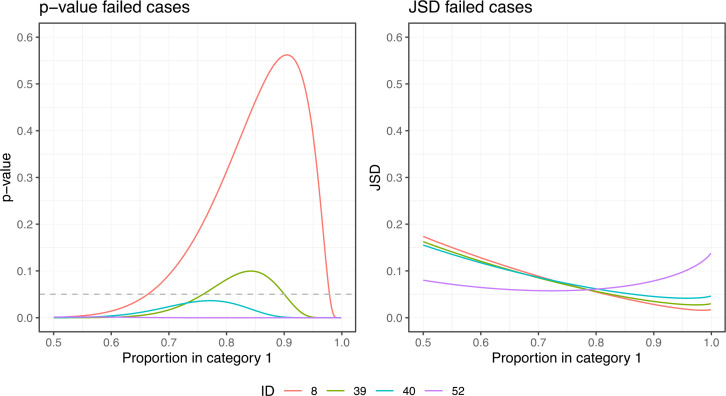
P-value of the chi-squared test (y-axes left) and JSD (y-axis right) between manually specified distributions with high probability in the first category score (x-axis) and EMA-data driven distributions.

**Figure S4:**
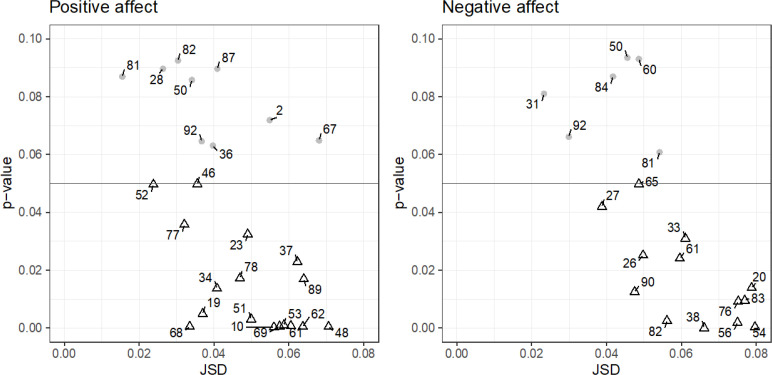
JSD values (x-axis) plotted against p-values (y-axis) of the chi-squared test for positive affect and negative affect. The numbers in the plot refer to participants’ ID numbers. The solid horizontal line denotes a p-value of .05. Black triangles denote that the p-value was lower than .05.

## Footnotes

1 Note that in this set-up, it can still not be established whether participants actually used the mean. We can only establish that the design pertained to the same summary statistic.

2 Subsequently, the authors computed correlations between these raw difference scores and self-reported well-being measures, and ran mediation analyses with self-reported resilience as a mediator between these raw difference scores and well-being measures. Finally, they ran cluster analyses on the raw difference scores, based on which they created groups for MANOVA analyses involving the well-being measures. For more details and the results of these analyses, we refer to the original study (Colombo et al., 2020).

3 For positive affect, the highest remaining difference between our calculation of the mean and the mean in the dataset was 0.038, and the second highest remaining difference was .002. For negative affect, the highest remaining difference was .029, and the second highest remaining difference was .000.

4 We used the interquartile range as a summary statistic of the EMA data rather than the standard deviation given that the EMA data were ordinal.

5 Note that theses ranges were expressed as integers (i.e., maximum score minus minimum score), while the RA summary scores were expressed to the decimal. This means that for example an RA summary score of 2.1 would fall outside of a range from 1 to 2.

6 In the original manuscript, the reported means for the difference scores were .074 for positive affect (SD = 0.85), and .415 (SD = .55) for negative affect.

## Action editor

Lars-Gunnar Lundh served as action editor for this article.

## Appendix A: Details analyses

### The optimization algorithm

In order to create the optimized RA probability distribution, we used an optimization algorithm (Chen, & Yin, 2016). Using this algorithm, we minimized the log-likelihood difference between the observed EMA distribution and the optimized RA distribution under a mean restriction (i.e., the RA summary).

The optimization algorithm struggled with probabilities that were exactly zero, as in this case the associated logits, which were used to ensure that the values remain positive, are minus infinity. A solution to deal with this problem is to add a constant to each category score. However, as the number of observations was relatively low, adding a constant may impact the estimated probabilities. Therefore, we used a two-step smoothing procedure. In the first step, we added a constant of 1 to each category score, and ran the optimization algorithm using the logits of these category scores as starting values. The final estimated logits in this procedure were then used as starting values for the second step of the analysis in which we added a constant of .1 to the observed category scores.

Using this procedure, the algorithm only failed in four cases for negative affect (ID = 8, 39, 40, 52). For all these cases, the RA summary was exactly 1 (i.e., the lowest category score), while their EMA measurements included values higher than 1. These cases could not be handled by the optimization algorithm, as the solution should ascribe a logit of infinity to category score 1, and minus infinity to the other category scores. However, for these cases there appears to be a clear solution, which is to ascribe near full probability to category score 1. We choose to ascribe near full probability rather than full probability in order to keep the probabilities similar to optimized probabilities, and in order to avoid having to divide by 0 for the chi-squared tests. We tried various configurations in which the highest probability was ascribed to the first reference category and the remaining probability was equally divided over the others and computed the difference tests for each (see Appendix B).

### Difference tests

The Chi-squared test is given by:

$$\chi_{d}^{2}=\sum \frac{\underline{\text{(}}{\underline{O}}_{\underline{i}}\underline{-}\underline{E}\underline{{}_{j}}{\underline{\text{)}}}^{\underline{2}}}{{\underline{E}}_{\underline{i}}}$$

in which *d* is the degrees of freedom, which were three in our case, as we imposed one restriction (i.e., the mean) on four probabilities that could be freely estimated. O and E refer to the number of observed counts and expected counts respectively per *i*th category score. We calculated the expected counts by multiplying the estimated probabilities by the total number of observations per participant.

The Jensen-Shannon divergence (JSD) is a smoothed and normalized version of the Kulback-Leibler divergence (KLD). The KLD is given by:

$$KLD(\text{P}∥\text{Q)=}\sum {}_{x\in X}P(x)\log\left(\frac{P(x)}{Q(x)}\right)$$

where x is the probability per category score for distributions P and Q. The JSD is then given by:

$$JSD(P∥\text{Q)=}\frac{1}{2}KLD(\text{P}∥\text{M)+}\frac{1}{2}KLD(Q∥\text{M),}$$

Where $M=\frac{1}{2}(P+Q)$

As can be seen from this equation, *M* is the average of the two probability distributions P and Q, and the JSD is the average of the KLD between probability distribution P and M and the KLD between probability distribution Q and M. When using the base-2 logarithm, the JSD ranges from 0 (identical) to 1 (completely different).

## Appendix B: Additional results

### Completed questionnaires

The mean number of completed questionnaires was 32.63 (*SD* = 6.03). The number of completed questionnaires for positive affect and negative affect was equal for all participants. Figure S1 presents a histogram of the number of completed questionnaires.

### Differences between step 1 and 2 in the optimization procedure

In order to keep the log-likelihood bounded during optimization, we added a constant to the observed category scores, using a two-step procedure (see Appendix A for details). In the first step we added 1 as a constant to each category score and ran the optimization algorithm with starting values based on these category scores. In the second step, we used the resulting estimated logits as starting values, while adding a constant of .1 tot the observed category scores. The differences between the estimated probabilities in step 1 and step 2 were small, as can be seen in Figure S2.

### Difference tests for failed cases

The optimization algorithm failed for cases that had a RA score of exactly 1 for negative affect. For these cases, we choose to manually ascribe high probability to the first category score, while keeping the other probabilities equal. The results of difference tests between these different distributions and their respective data-driven distribution can be found below in Figure S3. As can be seen from this figure, for two cases the p-value of the chi-squared test remained lower than .05 even for relatively low probability of category score 1 (ID = 40 & 52), while for the two others, a configuration was possible that resulted in a p-value that was higher than .05 (ID = 8 & 39). Therefore, the number of significant chi-squared tests was between 33 and 35.

### Comparison of p-values to JSD

To highlight participants who had high non-significant differences and small significant differences between observed EMA distributions and optimized RA distributions, we plotted their JSD value against their corresponding p-value in Figure S4. Note that in order to highlight values around a p-value of .05, these plots show only part of the total range of JSD values and p-values.
